# The genetic basis of adaptation to copper pollution in *Drosophila melanogaster*

**DOI:** 10.3389/fgene.2023.1144221

**Published:** 2023-04-04

**Authors:** Elizabeth R. Everman, Stuart J. Macdonald, John K. Kelly

**Affiliations:** ^1^ Molecular Biosciences, University of Kansas, Lawrence, KS, United States; ^2^ Center for Computational Biology, University of Kansas, Lawrence, KS, United States; ^3^ Ecology and Evolutionary Biology, University of Kansas, Lawrence, KS, United States

**Keywords:** heavy metals, toxicity, stress resistance, genome wide association, *Drosophila*

## Abstract

**Introduction:** Heavy metal pollutants can have long lasting negative impacts on ecosystem health and can shape the evolution of species. The persistent and ubiquitous nature of heavy metal pollution provides an opportunity to characterize the genetic mechanisms that contribute to metal resistance in natural populations.

**Methods:** We examined variation in resistance to copper, a common heavy metal contaminant, using wild collections of the model organism *Drosophila melanogaster*. Flies were collected from multiple sites that varied in copper contamination risk. We characterized phenotypic variation in copper resistance within and among populations using bulked segregant analysis to identify regions of the genome that contribute to copper resistance.

**Results and Discussion:** Copper resistance varied among wild populations with a clear correspondence between resistance level and historical exposure to copper. We identified 288 SNPs distributed across the genome associated with copper resistance. Many SNPs had population-specific effects, but some had consistent effects on copper resistance in all populations. Significant SNPs map to several novel candidate genes involved in refolding disrupted proteins, energy production, and mitochondrial function. We also identified one SNP with consistent effects on copper resistance in all populations near *CG11825*, a gene involved in copper homeostasis and copper resistance. We compared the genetic signatures of copper resistance in the wild-derived populations to genetic control of copper resistance in the *Drosophila* Synthetic Population Resource (DSPR) and the *Drosophila* Genetic Reference Panel (DGRP), two copper-naïve laboratory populations. In addition to *CG11825*, which was identified as a candidate gene in the wild-derived populations and previously in the DSPR, there was modest overlap of copper-associated SNPs between the wild-derived populations and laboratory populations. Thirty-one SNPs associated with copper resistance in wild-derived populations fell within regions of the genome that were associated with copper resistance in the DSPR in a prior study. Collectively, our results demonstrate that the genetic control of copper resistance is highly polygenic, and that several loci can be clearly linked to genes involved in heavy metal toxicity response. The mixture of parallel and population-specific SNPs points to a complex interplay between genetic background and the selection regime that modifies the effects of genetic variation on copper resistance.

## 1 Introduction

Heavy metals are naturally occurring elements enriched in some environments owing to industry and agriculture due to their usefulness as conductive materials (Karnchanawong and Limpiteeprakan, 2009) and as components of pesticides (Schutte et al., 1997; Roca et al., 2007). More than 5 million sites world-wide are polluted with dangerous levels of heavy metals and metalloids (He et al., 2015), and mismanagement of mining and industry waste as well as widespread pesticide use in agriculture are among the primary drivers of heavy metal pollution (Liu et al., 2018; Kang et al., 2020). When heavy metals become highly concentrated in soil and water, they pose a serious threat to ecosystem health (Li et al., 2019; Vareda et al., 2019). Additionally, because heavy metals are not biodegradable and persist and accumulate in contaminated sites (Sánchez-Chardi and Nadal, 2007; Ali et al., 2019; Ali and Khan, 2019), they can drive adaptation in populations. Indeed, variation in heavy metal resistance and adaptation has been linked to pollution in many species (e.g., Ashida, 1965; McNeilly, 1968; MacNair et al., 1993; Roelofs et al., 2006; Martins et al., 2007; Li et al., 2017; Kang et al., 2020; Li et al., 2021).

Numerous genes and pathways have the potential to contribute to metal resistance (Merritt and Bewick, 2017). For example, genes that regulate metabolism and homeostasis of biologically necessary heavy metals (such as copper and zinc) also interact with metals that have no biological role (such as cadmium and lead) (Olaniran et al., 2013; Calap-Quintana et al., 2017). Metallothioneins (Mtns), a small family of metal scavenger proteins present in prokaryotes and eukaryotes that likely arose through gene duplication events (Maroni and Watson, 1985; Moleirinho et al., 2011), fall into this category. Mtns primarily bind and sequester excess copper or zinc ions to reduce their intracellular concentration, but will also bind and thus help detoxify cadmium ions (Egli et al., 2003; Egli et al., 2006; Luo et al., 2020). Mtn sequence and expression variation has been previously linked to variation in metal resistance in insects and humans (Roelofs et al., 2006; Janssens et al., 2009; Adams et al., 2015; Catalán et al., 2016), suggesting they may play a role in adaptive responses to metal pollution.

Cytochrome p450s (Cyp450s), glutathione s transferases (Gsts), ABC-type transporters, and heat shock proteins (HSPs) all respond to a wide range of xenobiotic stressors and also have the potential to contribute to heavy metal resistance (Enayati et al., 2005; González-Guerrero et al., 2010; Gupta et al., 2010; Cao et al., 2017; Chain et al., 2019; Lu et al., 2021). For instance, in the mosquito *Aedes aegypti*, larval exposure to copper increased expression of cytochrome p450s, which ultimately led to greater resistance of larvae to insecticides (Poupardin et al., 2008). The potential for interplay between heavy metal pollution and chemicals used to reduce noxious mosquito populations has linked implications for both the evolution of the species as well as control of diseases that impact human populations (Poupardin et al., 2008; Riaz et al., 2009). Given the broad range of chemical stressors that induce them, Cyp450s, Gsts, HSPs, and Mtns have all been suggested as biomarkers of heavy metal pollution (Pyza et al., 1997; Tabrez and Ahmad, 2013; Saad et al., 2016) and both protein-coding and regulatory variation in or near these gene families could contribute to metal adaptation.

An important question is how frequently metal adaptation is conferred by a single major mutation as opposed to the aggregate effects of many loci. There are certainly well documented cases of adaptation to metals *via* mutations of large effect. For instance, populations of *Mimulus guttatus* growing in copper mine tailings in Copperopolis, CA, USA evolved copper resistance due to rapid evolution at the *Tol1* locus (MacNair, 1983; Wright et al., 2013). In *Saccharomyces cerevisiae*, copper resistance was linked to copy number variation of the copper chelating *CUP1* locus (Fogel and Welch, 1982; Karin et al., 1984). However, when variation in metal resistance has been examined using laboratory-derived populations the trait appears to be polygenic (Mahapatra et al., 2010; Ehrenreich et al., 2012; Zhou et al., 2016; Zhou et al., 2017; Evans et al., 2018; Everman et al., 2021), suggesting that single locus, large effect mutations that lead to adaptation may be rare. The fact that the laboratory populations used in most of these studies were naïve to metal stress motivated the present study: We examine multiple populations with varying exposure to metal pollution to better understand and characterize the genetic complexity underlying metal adaption in natural populations.

We characterized variation in copper resistance in multiple natural populations of *D. melanogaster* collected from sites influenced by local mining and agricultural activities. In addition, we characterized copper resistance in two independently derived wild-type *Drosophila* laboratory populations [*Drosophila* Synthetic Population Resource (DSPR) and the *Drosophila* Synthetic Reference Panel (DGRP)] to provide a comparison between our wild-collected populations and copper-naïve populations. We focused on copper because it is a common metal pollutant that is toxic at high concentrations (Olaniran et al., 2013; Zuily et al., 2022) but also one of the few heavy metals that is biologically essential (Calap-Quintana et al., 2017). Furthermore, Green et al. (2022) found that copper resistance varied in natural populations of *Drosophila melanogaster* and may be influenced by pollution associated with urbanization. *D. melanogaster* is an excellent model for assessing the impacts of copper pollution because populations are widely distributed in both contaminated and uncontaminated sites. In addition, there is extensive overlap of metal responsive genes between *D. melanogaster* and humans, and flies have been widely used as a model to characterize the genetic response to metal stress (Egli et al., 2006; Hua et al., 2011; Abnoos et al., 2013; Zhou et al., 2016; Calap-Quintana et al., 2017; Zhou et al., 2017; Everman et al., 2021).

We collected *D. melanogaster* from five sites that varied in heavy metal pollution level and found a positive association between copper resistance and the level of metal pollution at the site. We then used a pooled sequencing, bulked segregant analysis approach (Kelly and Hughes, 2019) to identify genetically variable loci associated with copper resistance in each population. We identified 288 SNPs and several potential candidate genes that were associated with variation in copper resistance. There was limited overlap in the genetic architecture of copper resistance in the wild-derived populations and the DGRP; however, overlap was more extensive between the wild-derived populations and the DSPR. Candidate genes included those that have been previously linked to copper metabolism, homeostasis, and toxicity as well as genes that represent novel candidates for copper resistance. By examining the genetic control of copper resistance in multiple wild-derived populations as well as against known copper-naïve DSPR and DGRP laboratory populations, we were able to identify loci that had similar effects across populations, as well as loci that had population-specific effects, which may depend on a combination of genetic background, selection intensity, and evolutionary history.

## 2 Materials and methods

### 2.1 Collections and fly rearing

**FIGURE 1:**
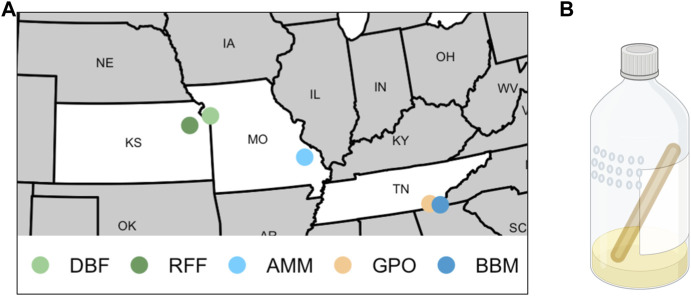
Sites and traps used to collect *Drosophila melanogaster*. **(A)** Flies were collected from two orchards (DBF = Duncan’s Berry Farm; RFF = Rees’ Fruit Farm), two mine sites (AMM = Anschutz Mine; BBM = Burra Burra Mine), and one open-air market (GPO = Gilliland Orchard). **(B)** A representation of 16oz plastic bottles baited with banana, yeast, and water that were used to collect flies (created with BioRender.com). Small holes (4 mm diameter) were melted into the sides of bottles in a grid pattern, and a 15 cm wooden craft stick was provided to prevent flies from sticking in the bait or the side of the bottle.

We collected flies from five field sites in the United States including two orchards (Duncan’s Berry Farm, DBF; Rees’ Fruit Farm, RFF), a small open-air market (Gilliland Orchard, GPO), and two mines (Anschutz Mine, AMM; Burra Burra Mine, BBM; Table 1 and Figure 1A). DBF is a small blueberry farm in Smithville, MO, USA and RFF is a fruit farm in Topeka, KS, USA that grows a variety of fruit including raspberries, apples, tomatoes, peaches, and apricots. Copper-containing pesticide use at the DBF and RFF sites is limited (personal comm. to ERE from DBF and RFF owners). The GPO site was in an open-air market in Cleveland, TN, USA, which does not have any on-site fruit production. During the collection period, the market was selling peaches grown in Hogback, SC, USA (∼240 km from the market, personal communication to ERE).

**TABLE 1:** Collection dates, number of *D. melanogaster* females collected at each site, and levels of replication for experiments.

Site	Site Location	Collection Dates	Tree Type	N Founder Females	Trait	N Individuals	Mean Females/Rep*
Duncan’s Berry Farm (DBF)	39.40 N, −94.57 W	Sept 3, 4, 19, 2018	apple, pear	100	CR	3,527	19.6
					SR	3,555	19.8
Gilliland Orchard (GPO)	35.15 N, −84.38 W	Jun 28–2 Jul 2019	oak, maple	63	CR	4977	19.9
					SR	998	19.9
Burra Burra Mine (BBM)	35.04 N, −84.38 W	Jun 28–2 Jul 2019	apple	89	CR	5004	20
					SR	1003	20
Anschutz Mine (AMM)	37.55 N, −90.28 W	Aug 12-16, 2019	honeysuckle	20	CR	1849	17.4
					SR	NA	NA
Rees’ Fruit Farm (RFF)	39.09 N, −95.59 W	Sept 27-30, 2019	apricot	784	CR	11,171	19.9
					SR	2002	20

Panel	Site Location	Collection Dates	Tree Type	N Strains	Trait	Mean Rep*/Strain	Mean Females/Rep*
DSPR	NA	NA	NA	194	CR	3.9	19.4
				1723	SR	Everman et al. (2019)	
DGRP	NA	NA	NA	152	CR	4.8	19.9
				168	SR	Everman et al. (2019)	

CR, copper resistance; SR, starvation resistance; *Replicate.

AMM (known historically as the Madison Mine) is an active cobalt mine in Fredericktown, MO, USA and is one of several mines located in the lead belt of Missouri. AMM currently encompasses 0.59 km^2^ and has been linked to metal contamination of soil and water at levels of environmental concern (Rangen and Mosby, 2015). A remedial investigation conducted in 2011 found elevated levels of several metals, including copper, in sediment of the nearby Little St. Francis River chat pile (3.5 km from collection site) (Rangen and Mosby, 2015). Flies were collected outside the Anschutz Mine premises less than 6 m from a flowing mine shaft (contaminated ground water exiting a mine shaft opening). BBM is a decommissioned copper mine which operated from 1899–1958 (Illo, 2009) and was the largest of three primary mines in the Copper Basin Mining District in Tennessee (Poste, 1932). BBM is currently listed as an non-NPL (National Priorities List) Superfund Site (EPA, 2011). Lee et al. (2002) reported elevated levels of copper in addition to zinc, cobalt, nickel, lead, and cadmium in creeks (North Potato watershed, David’s Mill Creek) that flow through the Copper Basin region. Flies were collected approximately 0.2 km from the Burra Burra mine pit.

We collected flies from wild populations daily for 3–6 days between June and September (DBF flies were collected in 2018, all other populations were collected in 2019; Table 1) using ten 16oz clear plastic bottle traps baited with half of one banana mixed with approximately one teaspoon of yeast and 10 mL water (Figure 1B). We melted 4 mm diameter holes into the top half of the bottles with one hole approximately every centimeter in a 3 by 6 grid (Figure 1B). A 15 cm wooden craft stick (e.g., Carolina Biological 971662) was placed in each trap to provide an area for flies to land. Traps were suspended with string from trees or bushes so that they remained in the shade and were replaced daily. Individual gravid females were aspirated from traps into cornmeal-molasses-yeast vials to lay eggs for 5 days to obtain males for species verification and to obtain flies to establish cage populations. Cage populations (30 cm × 30 cm × 30 cm, Bugdorm-1) were established for the DBF, GPO, and BBM collections using 20 virgin females and males from each site-specific founder *D. melanogaster* female. Given the large number of females collected from the RFF site, we used 5 virgin females and males to establish the RFF population cage to avoid overcrowding. Since only 20 females were obtained from the AMM site, we did not establish a population cage and instead maintained isofemale strains that were used to measure copper resistance.

We also assessed copper resistance in a subset of the *Drosophila* Synthetic Population Resource (DSPR, 194 strains, (King et al., 2012)) and the *Drosophila* Genetic Reference Panel (DGRP, 152 strains, (Mackay et al., 2012)). The DSPR and DGRP are two large panels of *D. melanogaster* inbred strains. These strains were included as representatives of heavy metal-naïve populations as they are derived from ancestral populations with low likelihood of exposure to heavy metals and have been maintained in standard laboratory conditions for at least a decade (the DSPR strains were derived from a global sampling of individuals collected before 1970 and were inbred by 2009; DGRP females were collected from an open-air farmer’s market in 2003 (Mackay et al., 2012)).

### 2.2 Phenotypic variation in stress tolerance

We assessed copper resistance (our focal trait) and starvation resistance (a representation of a non-metal stress trait) in the wild-derived flies in the third generation removed from the field (after one generation of mating freely in population cages) to limit the opportunity for lab adaptation. DBF, RFF, GPO, and BBM were measured as populations for both traits. AMM, DGRP, and DSPR were measured as isofemale/inbred strains for copper resistance only. Starvation resistance was measured previously in the DSPR and DGRP populations (Everman et al., 2019). We did not measure starvation resistance in the AMM population because this population had relatively few founder females and was not included in subsequent sequencing experiment. Females were used in all experiments. We focused on females to facilitate comparison with previously published studies and because females are the homogametic sex, which simplifies population genetic analyses. Experimental females were obtained from population cages by collecting embryos on apple juice agar plates baited with yeast paste (equal parts yeast and water). Apple juice plates were placed in the cages overnight and embryos were collected the following morning by suspending embryos in 1x PBS and rinsing them into a 50 mL conical tube. After the embryos settled to the bottom of the tube, 12 uL aliquots of embryos were distributed into cornmeal-molasses-yeast food vials. To obtain experimental females from the AMM isofemale strains and the DSPR and DGRP strains, we allowed adult females to oviposit in cornmeal-molasses-yeast vials for 2 days before removing all adults. All vials containing embryos underwent development in the same incubator at 25°C and ∼50% humidity on a 12:12 L:D cycle.

Flies were allowed to eclose for 2 days and then were transferred to new food vials for 24 h to ensure most if not all experimental females were mated. Experimental females were sorted from males into groups of 20 over CO_2_ anesthesia, placed in new cornmeal-molasses-yeast food vials, and were allowed to recover for 24 h. Following recovery, experimental females were transferred without CO_2_ into vials containing either 1.8 g Instant *Drosophila* Media (Carolina Biological Supply Company 173200) hydrated with 8 mL 50 mM Copper (II) Sulfate (CuSO_4_; Sigma-Aldrich C1297) or starvation media (1.5% agar with media preservatives prepared as described in Everman et al. (2019)). Copper and starvation resistance were assessed by counting the number of dead individuals every 24 h. When approximately 150 copper-exposed females from the DBF, RFF, GPO, and BBM populations remained alive, exactly 150 individuals from each population were collected and frozen for DNA extraction (described below). Counts of females tested for each trait and each population are summarized in Table 1.

We calculated copper and starvation resistance as the average time (in hours) until death per vial. Resistance measured in the AMM, DSPR, and DGRP populations is reported as average lifespan per strain. All phenotype residuals were close to normally distributed and our sample sizes are large, so we proceeded with parametric tests since our tests are likely robust to slight deviations from model assumptions. Variation in copper and starvation resistance was assessed separately with one-way analyses of variance (ANOVA), testing the effect of population on variation in average lifespan. We used Tukey’s HSD *post hoc* comparisons to test for significant differences among populations.

To determine whether copper and starvation resistance may be phenotypically correlated, we used a general linear model to test for a correlation between mean strain-specific measures of the two stress traits in the DSPR and the DGRP. We could not determine whether the two stress traits were correlated within each of the wild-derived populations because each individual fly is genetically distinct (whereas the DSPR and DGRP are comprised of many isogenic strains that consist of genetically identical individuals). Instead, we calculated the mean stress resistance for each population and used a general linear model to test for a correlation between mean population-level copper and starvation resistance. This second analysis allowed us to address whether variation in starvation resistance among wild-derived populations followed a pattern like that of copper resistance.

### 2.3 Genetic sample preparation and sequencing

We executed bulked segregant analysis (Michelmore et al., 1991; Pool, 2016; Kelly and Hughes, 2019) for the 4 wild-derived population cages (DBF, RFF, GPO, BMM), using a copper-resistant pool of 150 females (see above) and a matching control pool of 150 females randomly selected from the same generation of flies that were not exposed to copper. We made whole genome libraries for each of the eight pools of flies (copper-resistant and control pools of DBF, RFF, GPO, and BBM populations). DNA was extracted from batches of 15 females per pool using the Gentra Puregene kit (Qiagen) and then pooled at equal concentrations. Pool-specific barcoded libraries were generated using the NEBNext DNA Library Prep Reagent Set for Illumina (New England Biolabs) with size selection for 300-400bp inserts. Libraries were assessed with qPCR and were pooled at equimolar concentrations in the following combinations: library pool 1 = control and copper-resistant DBF pools; library pool 2 = control and copper-resistant RFF, GPO, and BBM pools). Paired-end 150bp reads from library pool 1 were sequenced over an NextSeq 550 High Output flowcell, resulting in 250x (DBF control pool) and 310x (DBF copper-resistant pool) coverage. Paired-end 150bp reads from library pool 2 were sequenced over an NextSeq2000 P2 flowcell, resulting in on average 131x coverage per pool (RFF control = 143x, RFF copper-resistant = 122x; GPO control = 124x, GPO copper-resistant = 124x; BBM control = 141x, BBM copper-resistant = 131x). Previous studies have demonstrated that use of sequencing data from multiple platforms does not introduce widespread systematic biases or issues related to variant calling (providing that similar platforms (Lam et al., 2012; Illumina, 2020) and the same quality filtering and variant calling procedures are used (Pan et al., 2022), see below). Library preparation and sequencing were completed at the University of Kansas Genome Sequencing Core.

### 2.4 Read mapping and variant calling

Quality assessment and trimming of reads was completed with fastp [v. 0.20.1 (Chen et al., 2018)] using a mean quality score of 30 over a window size of 5bp. Filtered reads were aligned to the *D. melanogaster* reference genome (Release 6.41) using bwa mem (v. 0.7.17-r1188 (Li and Durbin, 2009)) with default parameters. Aligned reads were sorted, read pairs were identified, and duplicates were marked and removed with samtools [v. 1.11 (Li et al., 2009; Danecek et al., 2021)]. We used bcftools mpileup and call (v. 1.11 (Danecek et al., 2021)) to generate a BCF file containing genotype likelihoods for the eight aligned BAM files and to call variants. vcfutils.pl varFilter was used to filter SNP and indel variants using default parameters, generating a final VCF file containing variants from all pools and retaining 1,986,553 variants (203,673 of which were indels). VCF files were filtered to remove indels and variable sites with more than two segregating bases and to retain variable sites on the 5 major *D. melanogaster* chromosome arms (2R, 2L, 3R, 3L, X) with a minimum read depth of 100 and with a minimum allele frequency of 0.05 in at least one of the four populations. Post filtering, 692,822 SNPs remained. Small differences in error rate between the NextSeq 550 and NextSeq 2000 may impact detection of low frequency variants (Davis et al., 2021), but since we eliminate the bulk of these we do not anticipate a major impact of sequencing platform on our results.

### 2.5 Genetic control of copper resistance

#### 2.5.1 Bulked segregant analysis in wild populations

To assess the shift in allele frequency at each variable site between the control and copper resistant pools of individuals, we first calculated the difference in arcsine square-root transformed allele frequency (z) between the two pools:

$$dz=z\left(Cu\right)-z\left(Avg\right)$$

where Cu and Avg represent copper-resistant and control pools (Walsh and Lynch, 2018; Kelly and Hughes, 2019). Positive *dz* values indicate that the reference base is more common in the copper-resistant pool than in the control pool.

We detected copper resistance SNPs by first determining the likelihood of the data assuming that the SNP has no effect on resistance (Model 0). In this case, dz will differ from zero only because of sampling error (the limited number of flies in each pool, finite sequencing depth). We next determined the likelihood allowing a real difference between the pools that is population specific (Model 4: the true dz is non-zero and is allowed to vary between populations). Treating Model 0 as the null model, we calculated a likelihood ratio statistic, LRT4, to test for copper resistance:

$$LRT4=2\left(LL_{4}-LL_{0}\right)$$

where $LL_{4}$ is the log-likelihood of the data under Model 4 (with four parameters estimated from the data) and $LL_{0}$ is the log-likelihood under Model 0 (no estimated parameters). Comparison to a chi-square distribution with 4 degrees of freedom yields a *p*-value. Given the *p*-values from all SNPs, positive loci were identified using a Benjamini–Hochberg corrected false discovery rate (FDR) of 10%. The 10% FDR threshold was used as a permissive filter prior to the testing of additional LRT models. We chose a permissive threshold to avoid failing to detect signatures of selection or overlap in genetic architecture between populations solely due to an overly strict significance threshold, accepting that this likely increases our false positive rate.

**FIGURE 2:**
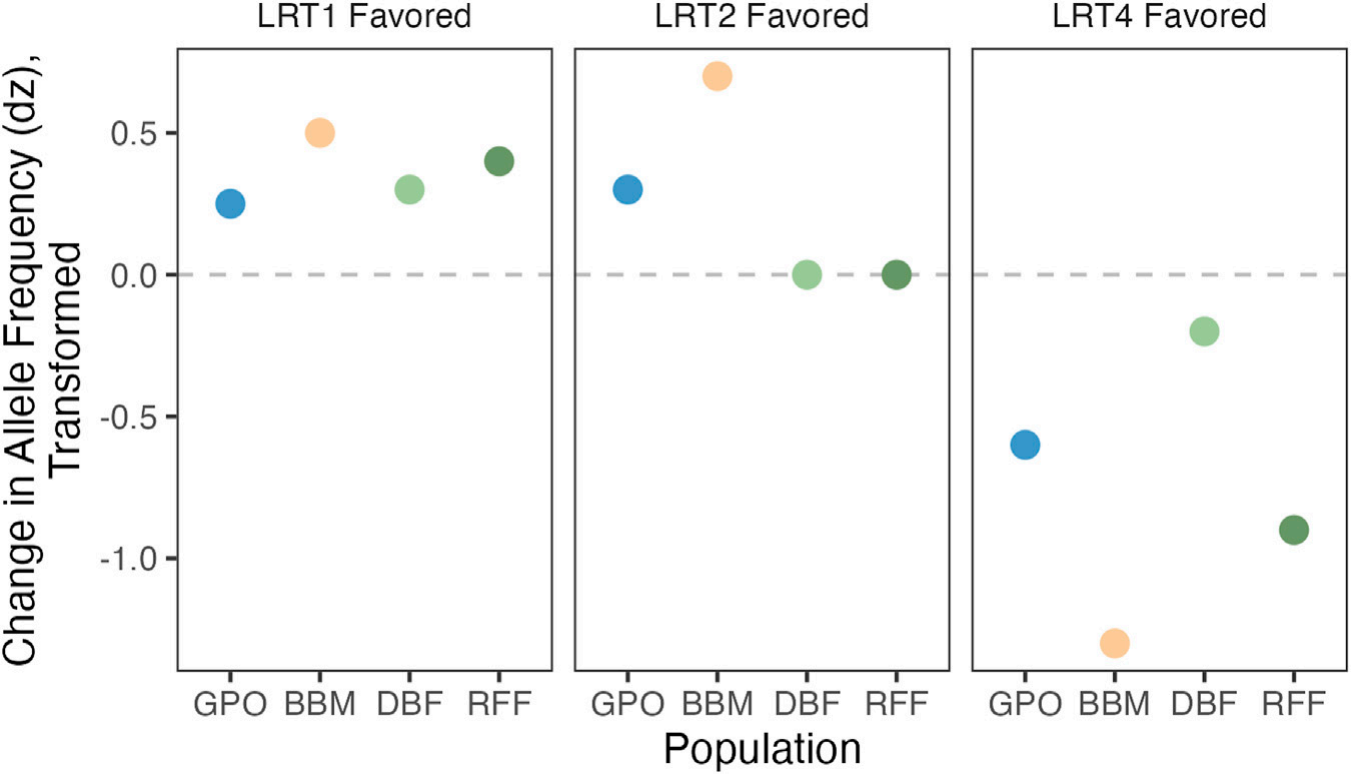
Conceptual representation of LRT models for significant SNPs. SNPs that shift in allele frequency between control and copper-resistant pools *in the same way* will give the lowest *p*-values using LRT1. LRT2 should most effectively identify parallel shifts in allele frequency between the two high copper resistance populations (BBM and GPO) versus the low copper resistance populations (DBF and RFF). Shifts in allele frequency that are population-specific will yield the lowest *p*-values using LRT4.

LRT4 is fully general and does not distinguish between different patterns of allele frequency shift between pools. To refine our estimates for SNP effects, we fit two additional models (Models 1 and 2) that constrain responses across populations (see Figure 2; Table 2). The simplest case (Model 1) posits a strictly parallel response: The true dz is the same across populations and can be characterized with a single parameter. We tested Model 1 against the null using:

$$LRT1=2\left(LL_{1}-LL_{0}\right)$$

which is compared to the chi-square distribution with 1 df. Model 2 is a generalization of Model 1 and allows the parallel shift to differ in magnitude between two groups, populations with high copper exposure risk (BBM and GPO) and populations with low copper exposure risk (RFF and DBF). We tested this two-parameter model versus the null hypothesis using:

$$LRT2=2\left(LL_{2}-LL_{0}\right)$$

which is compared to the chi-square distribution with 2 df. To determine the best fitting model for each SNP initially identified as significant by LRT4, we compared the raw *p* values from the three LRT models (LRT1, LRT2, and LRT4; named using the number of parameters in each test) and assigned the model resulting in the lowest *p*-value. Five SNPs showed identical *p* values for more than one model, and in these cases we assigned the model yielding the highest log-likelihood value.

**TABLE 2:** Summary of LRT models.

Test statistic	Allele frequency shift	Comparison	Model parameters	N SNPs
LRT1	Parallel shift in multiple populations	LL_0_	1	70
LRT2	Parallel shift in populations with similar exposure risk	LL_0_	2	23
LRT4	Population-specific shift in allele frequency	LL_0_	4	195

#### 2.5.2 Population and pool differentiation

We used allele frequency estimates from the control pools of the DBF, RFF, GPO, and BBM populations to test for population differentiation using global F_ST_ values calculated with the R package poolfstat [v. 2.1.1 (Hivert et al., 2018)]. We also estimated F_ST_ for each SNP to identify regions of the genome that were enriched for SNPs that contribute to population differentiation. SNP-specific F_ST_ estimates were compared to the results of our bulked segregant analysis (BSA) to infer the history of natural selection on putative copper resistance loci.

#### 2.5.3 Genetic characterization of copper resistance in the DGRP

The DGRP is a widely used *Drosophila* mapping population, and the full details on the generation, sequencing, and genome-wide association (GWA) mapping can be found from Mackay et al. (2012); Huang et al. (2014). The structure of the DGRP is distinct from the wild-collected populations examined in this study because it consists of fully inbred isogenic strains, allowing us to use GWA mapping to identify variants that are associated with copper resistance. The online DGRP2 GWA mapping tool (http://dgrp2gnets.ncsu.edu (Mackay et al., 2012)) accounts for *Wolbachia* infection and inversion status of the DGRP lines in the calculation of test statistics used to determine probabilities that the approximately 4 million variable sites are associated with phenotypic variation (Mackay et al., 2012; Huang et al., 2014). We assessed significance using a 5% false discovery rate threshold as well as the liberal threshold common in DGRP studies (*p* < 10^−5^) that is used because GWA using <200 lines is underpowered to detect variants with effects typical of highly polygenic traits (Vaisnav et al., 2014; Huang et al., 2020). The more permissive threshold (*p* < 10^−5^) facilitates comparison of our results with previously published studies and helps detect SNPs with true effects on copper resistance; however, the false positive rate is also inflated, so our results should be interpreted with this tradeoff in mind.

We estimated broad sense heritability for copper resistance in the DGRP by first estimating genetic and phenotypic variance from linear mixed model using the lme and varcomp functions in R (APE (Paradis et al., 2004), nlme (Pinheiro et al., 2017)) and then calculating the proportion of total variance explained by estimated genetic variance (Lynch and Walsh, 1998).

### 2.6 Overlap in the genetic control of copper resistance across populations

We assessed overlap in the genetic control of copper resistance in the DGRP with signals of copper-associated loci from our wild-derived populations (DBF, RFF, GPO, BBM) by comparing significant DGRP loci (*p* < 10^−5^) with those that shifted in allele frequency between the control and copper-resistant pools (significant for LRT1, LRT2, or LRT4). For SNPs that were within or near genes [within 3,000 bp, position information obtained from FlyBase (Thurmond et al., 2019)], this comparison was made at the gene level. For the remaining SNPs not associated with genes, and because linkage disequilibrium is low in *D. melanogaster*, we assessed overlap at the SNP level after converting DGRP SNP position annotation from Release 5 to Release 6 (Mackay et al., 2012; Thurmond et al., 2019). We also assessed overlap between copper-associated loci from the four wild-derived populations and previously resolved QTL intervals identified in the DSPR (Everman et al., 2021) by identifying QTL intervals within which BSA SNPs were found.

### 2.7 Gene ontology analysis

We carried out GO analysis using the Bioconductor annotation package org. Dm.eg.db (v. 3.14.0) (Carlson, 2019) and the R package GOstats (v. 2.60.0) (Falcon and Gentleman, 2007), which employs hypergeometric tests for overrepresentation of GO terms followed by multiple test correction. We obtained annotation and ontology information for the lists of genes using the *D. melanogaster* annotation tool available from biomaRt (v. 2.50.3 (Durinck et al., 2005)) *via* Ensembl (Cunningham et al., 2022) and the org.DM.eg.db R package.

### 2.8 Data availability

Starvation resistance data for the DSPR and DGRP are available from Everman et al. (2019). Adult female 48-h copper resistance data for the DSPR are available from Everman et al. (2021). Phenotype data generated in this study are available from FigShare. All code and allele frequency data are available from accompanying Supplementary Materials deposited on FigShare (https://doi.org/10.6084/m9.figshare.22332775.v1). Sequence data are available from NCBI SRA BioProject PRJNA923720.

## 3 Results

### 3.1 Copper resistance is highly variable in wild-derived and laboratory populations

**FIGURE 3:**
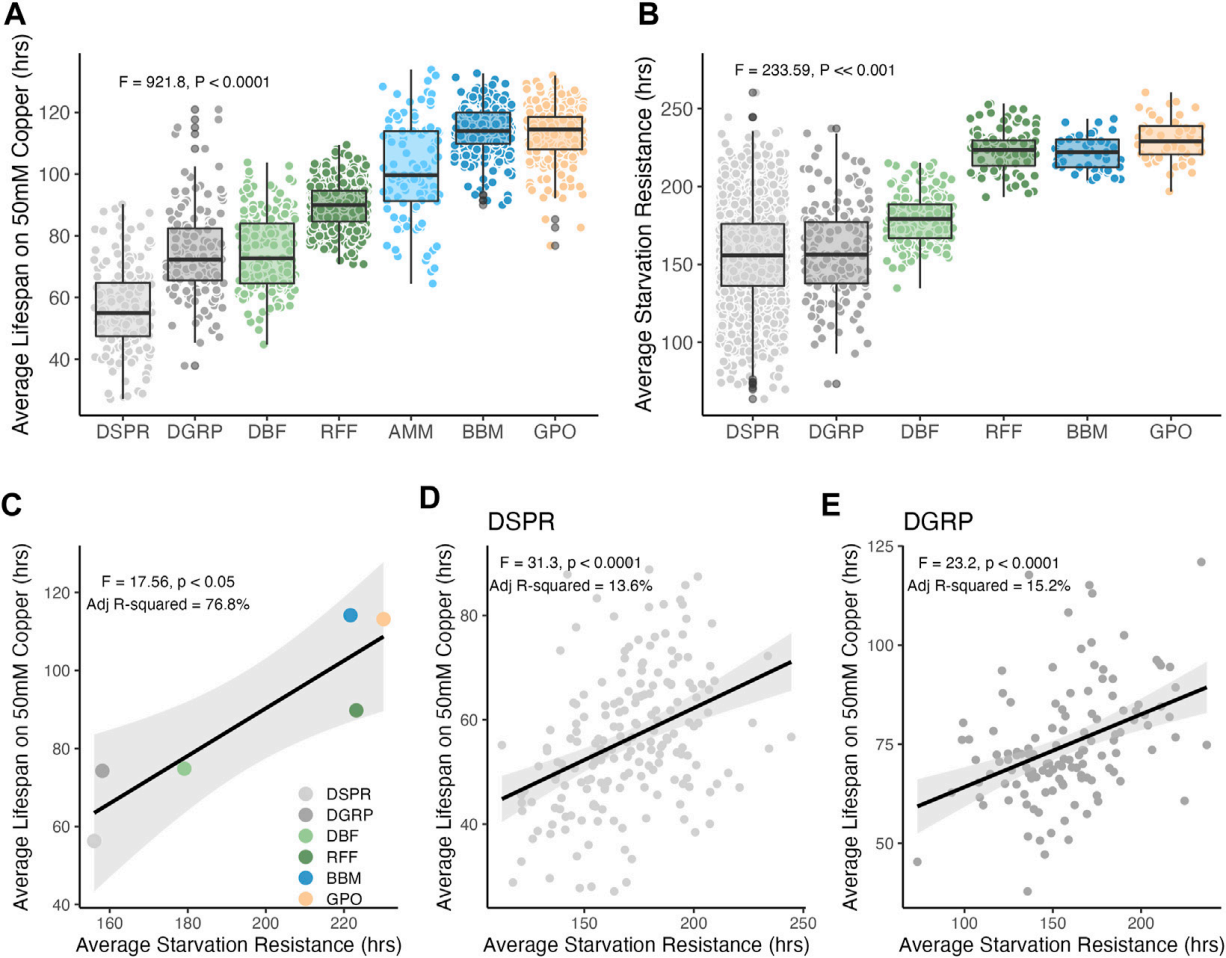
Copper and starvation resistance vary within and among populations. **(A)** Copper resistance significantly varied among populations (F_6,1685_ = 921.8, *p* < 0.0001), following a trend where resistance was higher in populations with closer proximity to sources of heavy metal contamination. Tukey’s HSD *post hoc* comparisons revealed that copper resistance was significantly different in all populations (adj *p* < 0.0001) except for the DGRP and DBF populations (adj *p* = 0.9) and the BBM and GPO populations (adj *p* = 0.9). **(B)** Starvation resistance also varied within and among populations. Tukey’s HSD *post hoc* comparisons revealed that starvation resistance was not different between the two laboratory populations (DSPR vs. DGRP, adj *p* = 0.94) or RFF, BBM, and GPO (all pairwise comparisons adj *p* > 0.64). Starvation resistance in the DBF population was significantly higher than the DSPR and DGRP and significantly lower than the other wild-derived populations (all pairwise comparisons adj *p* < 0.0001). In A and B, each point indicates either the strain-level mean (DSPR and DGRP, Table 1) or vial mean (N ≈ 20 females) resistance. **(C)** Population-level mean copper and starvation resistance were correlated with mean starvation resistance (F_1,4_ = 17.6, *p* = 0.014). **(D)** Starvation and copper resistance were correlated among DSPR strains (F_1,191_ = 31.3, *p* < 0.0001, adj *R* ^2^ = 13.6%) as well among DGRP strains (E. F_1,122_ = 23.2, *p* < 0.0001, adj *R* ^2^ = 15.3%). In **(D, E)**, the black line shows the best fit line and shading indicates the 95% CI of the regression model.

Adult female copper resistance (measured as average lifespan on copper containing food) was highly variable within and between all populations, with average lifespan ranging from 56.3 ± 0.95 s. e. to 114.2 ± 0.50 s. e. hours (Population: F_6,1685_ = 921.8, *p* < 0.0001; Figure 3A). Average copper resistance was also distinct in nearly every population; the only populations with similar levels of copper resistance were the DGRP and DBF populations (DBF vs. DGRP: adj *p* = 0.99) and the BBM and GPO populations (BBM vs. GPO: adj *p* = 0.94). All other pairwise comparisons of average copper resistance were significant (Tukey’s HSD; adj *p* < 0.0001; Figure 3A). Overall, average copper resistance was lowest in the DSPR laboratory population and highest in the wild-derived BBM and GPO populations (adj *p* < 0.0001; Figure 3A).

Figure 3A shows variation in copper resistance that is consistent with selection for resistance to heavy metals, but this pattern could also be explained by variation in overall stress resistance among the populations. To address this, we measured starvation resistance in four of the wild-derived populations (DBF, RFF, BBM, GPO) and leveraged previously-published starvation data from the DSPR and DGRP laboratory populations (Everman et al., 2019). We treated starvation resistance as a representative non-metal stress trait that could contribute to copper resistance (i.e., through initial avoidance of copper-containing food). Starvation resistance was variable within and among populations (F_5,2265_ = 233.6, *p* < 0.0001; mean starvation resistance: 156.0 ± 0.71 s. e.–230.1 ± 1.9 s. e. hours; Figure 3B). However, population-level differences in starvation resistance were less distinct than for copper resistance. Variation in starvation resistance among the DSPR and DGRP strains nearly encompassed the range of starvation resistance observed for all other populations (Figure 3B). In contrast, variation in copper resistance in wild-derived populations extended beyond the range of copper resistance observed in either laboratory population (Figure 3A). Generally, average starvation resistance was significantly different between the laboratory and wild-derived populations, with the exception of DBF which was intermediate to the laboratory populations and BBM, RFF, and GPO. Among all populations, mean copper and starvation resistance were correlated (F_1,4_ = 17.6, *p* = 0.013, adj *R* ^2^ = 76.8%, Figure 3C). However, the correlation between strain-specific copper and starvation resistance in the laboratory populations was modest, suggesting that starvation resistance explains less than 20% of variation in copper resistance when genetic variation can be controlled for (DSPR: F_1,191_ = 31.3, *p* < 0.0001, adj *R* ^2^ = 13.6%, Figure 3D; DGRP: F_1,122_ = 23.2, *p* < 0.0001, adj *R* ^2^ = 15.2%; Figure 3E). These pattens suggest that variation in copper resistance among populations is not solely due to overall patterns of stress resistance in the wild-derived populations.

### 3.2 Copper resistance is influenced by multiple loci in wild-derived populations with both consistent and population-specific effects

**FIGURE 4:**
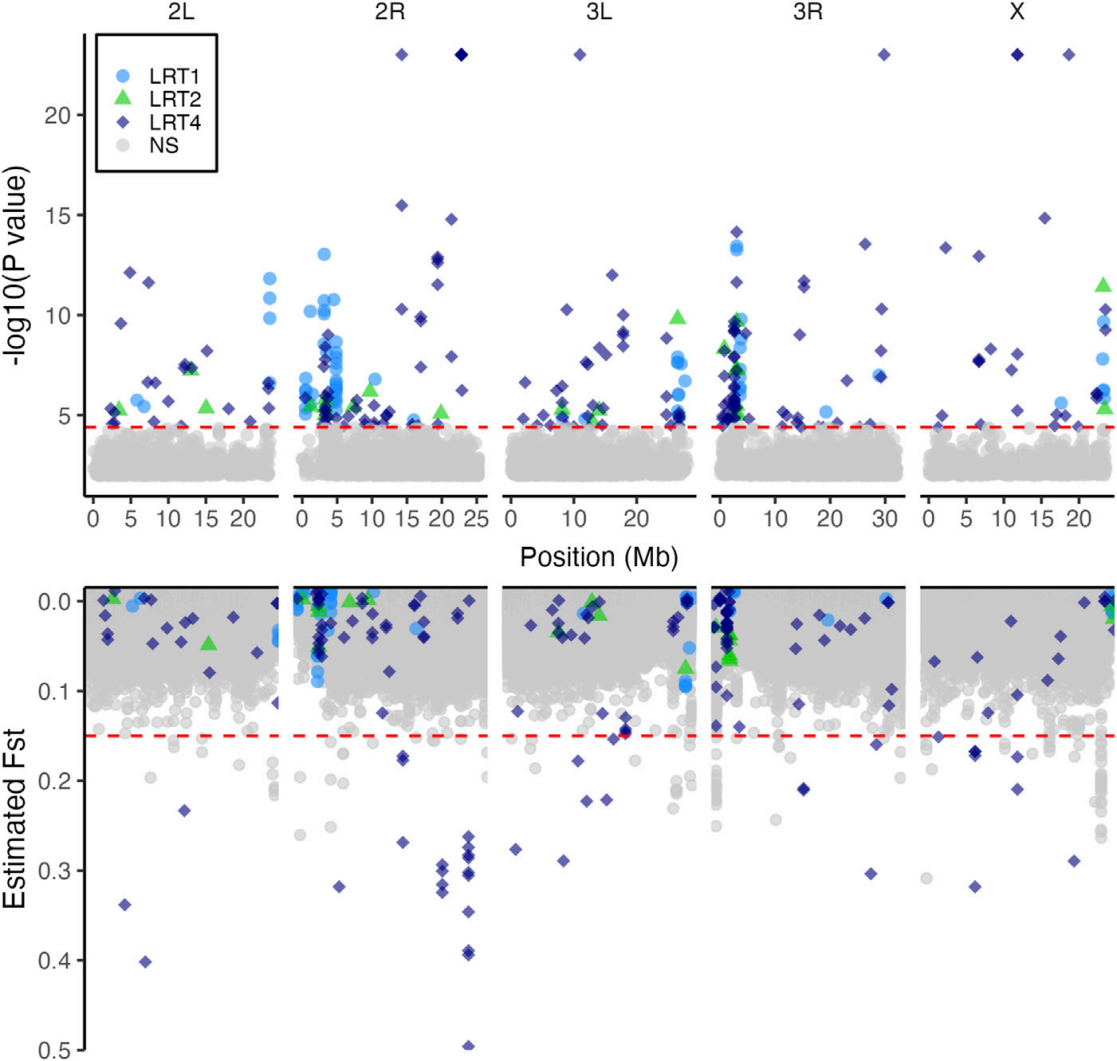
Copper resistance is influenced by multiple loci in wild-derived populations. The top panel shows 288 SNPs which shifted in allele frequency in a manner consistent with LRT1 (parallel shift, 70 SNPs), LRT2 (similar shift in high vs. low copper resistance populations, 23 SNPs), or LRT4 (population-specific shift, 195 SNPs). Points are partially transparent to show overlap of multiple SNPs. The bottom panel shows estimated F_ST_ values for each SNP. In both panels, SNPs are shaded according to which LRT model best fit their shift in allele frequency between the average and high copper resistance pools. 39 SNPs with population-specific effects on copper resistance (LRT4) also had elevated F_ST_ values, suggesting that these SNPs also contribute to population differentiation. In both plots, red horizontal dashed lines indicate thresholds; in the top panel the threshold indicates the 10% FDR cutoff used to identify significant SNPs. In the bottom panel the threshold is set at F_ST_ = 0.15 to differentiate SNPs with elevated F_ST_ estimates (Frankham et al., 2002).

We applied the bulked segregant analysis (BSA) tests to 692,822 SNPs. The most general model LRT4 identified 288 SNPs associated with copper resistance (Figure 4, top panel). Of these, LRT1, which tested for parallel shift in allele frequency in multiple populations, was chosen as the best fitting model for 70 SNPs. LRT2, which tested for a correspondence between exposure risk of populations and allele frequency shift, was the best fitting model for 23 SNPs. The remaining 195 SNPs were best fit by LRT4 indicating population-specific shifts in allele frequency between the copper-resistant and control pools (Figure 4; Table 2).

Of the 288 SNPs significantly associated with copper resistance, 198 (68.8%) fell within 103 distinct genes or within 3000bp of 120 distinct genes (Table S1). Because a small number of SNPs fell within multiple overlapping genes, the total number of genes containing or near LRT-associated SNPs was 220. The remaining 90 SNPs were further than 3000bp from any gene. Forty-seven genes have more than one SNP within or near them, with a maximum of 31 SNPs falling within a single gene (*Pzl*; Supplementary Figure S2, Supplementary Table S1). Gene ontology analysis revealed enrichment of genes involved in a wide range of categories which included several related to mitochondrial function (Supplementary Figure S2). We did not observe enrichment of genes specifically related to metal homeostasis, detoxification, or toxicity.

We examined the distribution of maximum LRT values for each model as they varied across the genome in 100 kb intervals and found that higher maximum LRT values were more common near the centromere regions of chromosome 2R and 3R (Supplementary Figure S3). SNPs with significant LRT values tended to co-localize in peaks in these regions as well (Supplementary Figure S3). This finding is consistent with a previous study of parallel selection in *Drosophila simulans* (Kelly and Hughes, 2019), and supports patterns expected for regions of low recombination rates.

### 3.3 Wild-derived populations are genetically distinct with regions of elevated F_ST_

We calculated pairwise F_ST_ using allele frequencies estimated from the control pools of each natural population for the 692,822 SNPs to measure differentiation among the wild populations. Overall, global F_ST_ was low (F_ST_ = 0.005 ± 9.06 × 10^−5^), consistent with previous studies of genetic differentiation in *D. melanogaster* populations (Ives, 1945; Verspoor and Haddrill, 2011; Mateo et al., 2018). While low, pairwise F_ST_ estimates were significantly positive indicating that the four wild-derived populations are genetically distinct (Table 3).

**TABLE 3:** Pairwise genome-wide F_ST_ values.

	DBF	BBM (se)	GPO (se)	RFF (se)
DBF	NA	0.004 (0.0001)	0.008 (0.0002)	0.0009 (0.0001)
BBM	-	NA	0.007 (0.0001)	0.003 (0.0001)
GPO	-	-	NA	0.007 (0.0001)
RFF	-	-	-	NA

Using a threshold of F_ST_ > 0.15 to indicate moderate to significant differentiation (Hartl and Clark, 1997; Frankham et al., 2002), SNP-wise F_ST_ estimates revealed 142 SNPs distributed across the five major chromosome arms that contribute to population differentiation (Figure 4, lower panel). Of these, 93 SNPs fell within genes (85 SNPs) or were within 3000bp of genes (8 SNPs) (Supplementary Table S2). Gene ontology analysis revealed enrichment of genes belonging to a diverse set of categories including “response to chemical” (GO term ID: 0042221) and others related to mitochondrial function and protein folding (Supplementary Figure S2). As with the BSA results, we did not observe any enrichment of SNPs with elevated F_ST_ in genes directly related to copper response. Like our BSA results, SNPs with high F_ST_ estimates tended to co-localize in peaks near centromeres and telomeres (Figure 4), and 39 of these SNPs were among those associated with copper resistance.

Notably, SNPs that were significantly associated with copper resistance in our BSA results (above) exhibited a tenfold increase in F_ST_ estimates relative to non-significant SNPs (LRT-significant SNPs average F_ST_ = 0.061 ± 0.006 s. e.; Non-significant SNPs F_ST_ = 0.005 ± 0.00002 s. e.). High F_ST_ of significant SNPs was largely driven by the farm-mine contrasts (LRT-significant SNPs average F_ST_ = 0.055) as the farm-farm contrasts resulted in lower levels of differentiation at significant sites (LRT-significant SNPs average F_ST_ = 0.033). In addition, BSA-SNPs with elevated Fst estimates usually had population-specific responses to copper resistance (i.e., they were most significant for LRT4; Figure 4, Table S1). The F_ST_ estimates corroborate the BSA results because they suggest a history of selection acting on loci that are associated with copper resistance.

### 3.4 Copper resistance is influenced by multiple loci in the DGRP

**FIGURE 5:**
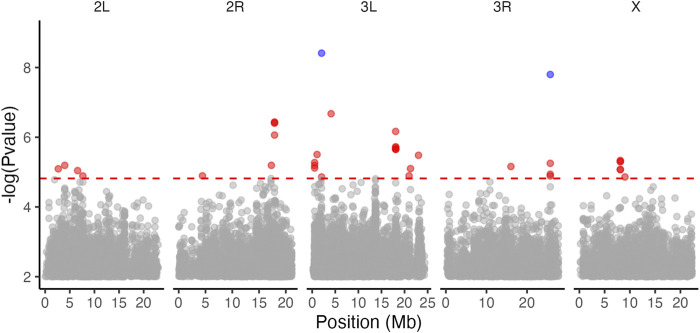
Genome wide association mapping of copper resistance in the DGRP identified multiple significantly associated SNPs. The red dashed line indicates *p* < 10^−5^ and variants surpassing this value are highlighted in red. Sites that were significantly associated with copper resistance at a 5% FDR are highlighted in blue.

Heritability of copper resistance was high in the DGRP (H^2^ = 84.9%). Using genome-wide association mapping, we identified two SNPs associated with copper resistance in the DGRP at a 5% FDR that were in introns of *sls* and *CG31038* (Figure 5). Neither gene has previously been associated with copper resistance. Using the more relaxed significance threshold that is typically applied to the DGRP (*p* < 10^−5^) (Mackay et al., 2012; Huang et al., 2014; Morozova et al., 2014; Zhou et al., 2016), we found 35 SNPs associated with copper resistance. The SNPs were near or within 17 annotated genes, with some genes containing multiple SNPs (Supplementary Table S3). None of the 17 genes have been previously associated with copper, heavy metal resistance, or responses to stress, and there was no overlap at the SNP or gene level between the DGRP and wild-derived population results.

### 3.5 Overlap in the genetic control of copper resistance between laboratory and wild-derived populations

We examined overlap between our BSA results and the SNPs associated with copper resistance in the DGRP at the gene level for all SNPs that fell within or near (within 3000bp of) genes and at the SNP level for all SNPs that could not be linked to genes. We found that GWA in the DGRP highlighted a set of genes and SNPs that were entirely distinct from the genes and SNPs associated with copper resistance in the wild-derived populations. The closest DGRP and BWA SNPs were >30,000 bp apart.

Overlap between our BSA results and regions associated with copper resistance in the DSPR was more extensive. Previous assessments of copper resistance carried out in the DSPR multi-parental mapping population indicated that allelic variation in several regions of the genome was linked to variation in copper resistance (Everman et al., 2021). This previous work highlighted several candidate genes involved in copper binding and metabolism and suggested that natural variation in genes involved in copper homeostasis may be under selection in natural populations. We identified one SNP upstream of the gene *CG11825* that was highlighted by QTL mapping in the DSPR (Q5 in that study), but no other candidate genes identified in that work contained or were near SNPs associated with copper resistance in our wild-derived populations. Nine of the 12 QTL regions identified in our previous work (Everman et al., 2021) spanned regions that contained a total of 31 SNPs with significant LRT values. All but one of the 31 SNPs that fell within QTL was in or near an annotated gene, and several genes were identified as potential candidate genes in the present study including *blw*, *ATPsynβL*, *CG7430*, and *LRR* in addition to *CG11825* (discussed below).

SNPs associated with copper resistance in the DGRP overlapped with two of the previously identified DSPR QTL. One DGRP SNP located on chromosome arm 2R fell within the Q5 DSPR QTL interval and six DGRP SNPs on chromosome arm 3L fell within the Q9 DSPR QTL interval. Combined, the DSPR Q5 and Q9 intervals contained several candidate copper resistance genes, which were followed up with RNAi in our previous work (Everman et al., 2021). However, none of the DGRP SNPs were near any of these functionally tested candidate genes.

Our assessment of overlap between the genetic architecture of copper resistance previously reported in the DSPR and the populations examined in this study may be influenced by differences in how the copper resistance traits were measured. In the present study, we measured copper resistance as adult female lifespan and in our previous work, DSPR copper resistance was assessed as survival after 48 h of exposure. However, for a subset of 194 DSPR strains, these two measures are highly correlated (F_1,192_ = 315.1, *p* << 0.0001, adj *R* ^2^ = 62%, r = 80%; Supplementary Figure S4). This correlation suggests that it is unlikely that the overlap in genetic architectures of copper resistance between the DSPR and the other populations assessed in this study is solely due to subtle differences in the phenotype assayed.

## 4 Discussion

### 4.1 Copper resistance in wild-derived populations is associated with historical heavy metal exposure

We demonstrate a strong association between copper resistance within a population and its proximity to sources of pollution. Flies collected from smaller fruit farms (DBF and RFF) where copper-containing pesticide use is limited had lower levels of copper resistance, whereas flies collected from mining sites (AMM and BBM) had higher levels of copper resistance (Figure 3A). We note that GPO–an open-air fruit market specializing in peaches–yielded flies with copper resistance similar to that of flies from the BBM site (Figure 3A). Dispersal estimates for *D. melanogaster* suggest that flies can disperse up to 12 km and possibly further if assisted by wind (Leitch et al., 2021). Despite the proximity of these collection sites in the Copper Basin region of Tennessee (∼43 km apart; Figure 1A), pairwise population differentiation estimates suggest that the BBM and GPO populations are distinct (Table 3). Further, because produce sold at the GPO site is grown in Hogback, South Carolina, USA, it is likely that the high levels of copper resistance in the BBM and GPO wild-derived populations are influenced by distinct sources of selection. Copper-containing fungicides are often used to control infections caused by *Taphrina deformans* in peach trees (Pecknold, 2015; Reis et al., 2021), as well as fungi and bacteria that damage wine grapes, olives, and citrus (Schutte et al., 1997; Roca et al., 2007; Peña et al., 2018), increasing the copper exposure risk for flies collected from the large commercial orchards such as the GPO site. Use of copper-containing pesticides may contribute to elevated levels of copper resistance in the GPO flies, whereas elevated copper resistance in the BBM flies is more likely to be due to historical mining activities.

The high level of copper resistance in flies descended from the BBM population is striking because no mining activity has occurred at Burra Burra Mine for more than 60 years. Heavy metal pollution persists in contaminated soil and water sources for many years (Suman et al., 2018; Ali et al., 2019) and tends to accumulate through food chains, becoming increasingly concentrated at higher trophic levels (Heikens et al., 2001; Sánchez-Chardi and Nadal, 2007; Gall et al., 2015; Ali et al., 2019; Ali and Khan, 2019). We did not measure levels of copper or other heavy metals in soil or plant material at the collection site, but our results suggest that sources of selection leading to high copper resistance continue to persist at Burra Burra Mine.

Mining activity is also the most likely environmental contributor to high copper resistance in the flies collected from Anchutz Mine (AMM) (Figure 3). Although copper is not the primary product of the mine, exposure to toxic levels of other heavy metals may drive the evolution of increased copper resistance as a correlated response to selection. For example, experimental evolution of the least killifish *Heterandria formosa* in response to cadmium stress also increased resistance to copper toxicity (Xie and Klerks, 2003). Studies using laboratory populations have demonstrated overlap in the genetic control of resistance to cadmium and copper in *Caenorhabditis elegans* (Evans et al., 2018) and cadmium and lead in *D. melanogaster* (Zhou et al., 2017). More generally, many genes involved in the metabolism of copper and response to copper toxicity are known to interact with other heavy metals (Egli et al., 2006; Yepiskoposyan et al., 2006; Adams et al., 2015; Calap-Quintana et al., 2017). Our intriguing results suggest that a broader sampling of populations associated with mines is warranted and would deepen our understanding of the historical selection pressures exerted on *D. melanogaster* populations and of the potential evolutionary consequences metal pollution presents to other species.

### 4.2 Genetic control of copper resistance is complex and suggests novel candidate genes

The genetic control of shifts in resistance to heavy metals has been examined in several species, and instances of both single locus (e.g., Wright et al., 2013; 2015) and polygenic control of resistance have been characterized (e.g., Ehrenreich et al., 2012; Evans et al., 2018). We identified 288 SNPs distributed across the genome that contribute to copper resistance in the wild-derived populations (Figure 4, Table S1) and 35 SNPs associated with copper resistance in the DGRP laboratory population (Figure 5). Polygenic control of copper resistance is consistent with previous findings from the DSPR indicating that copper resistance has a complex genetic basis (Everman et al., 2021) and from work carried out in other laboratory populations that may be naïve to metal stress (Ehrenreich et al., 2012; Zhou et al., 2016; Zhou et al., 2017; Evans et al., 2018).

**FIGURE 6:**
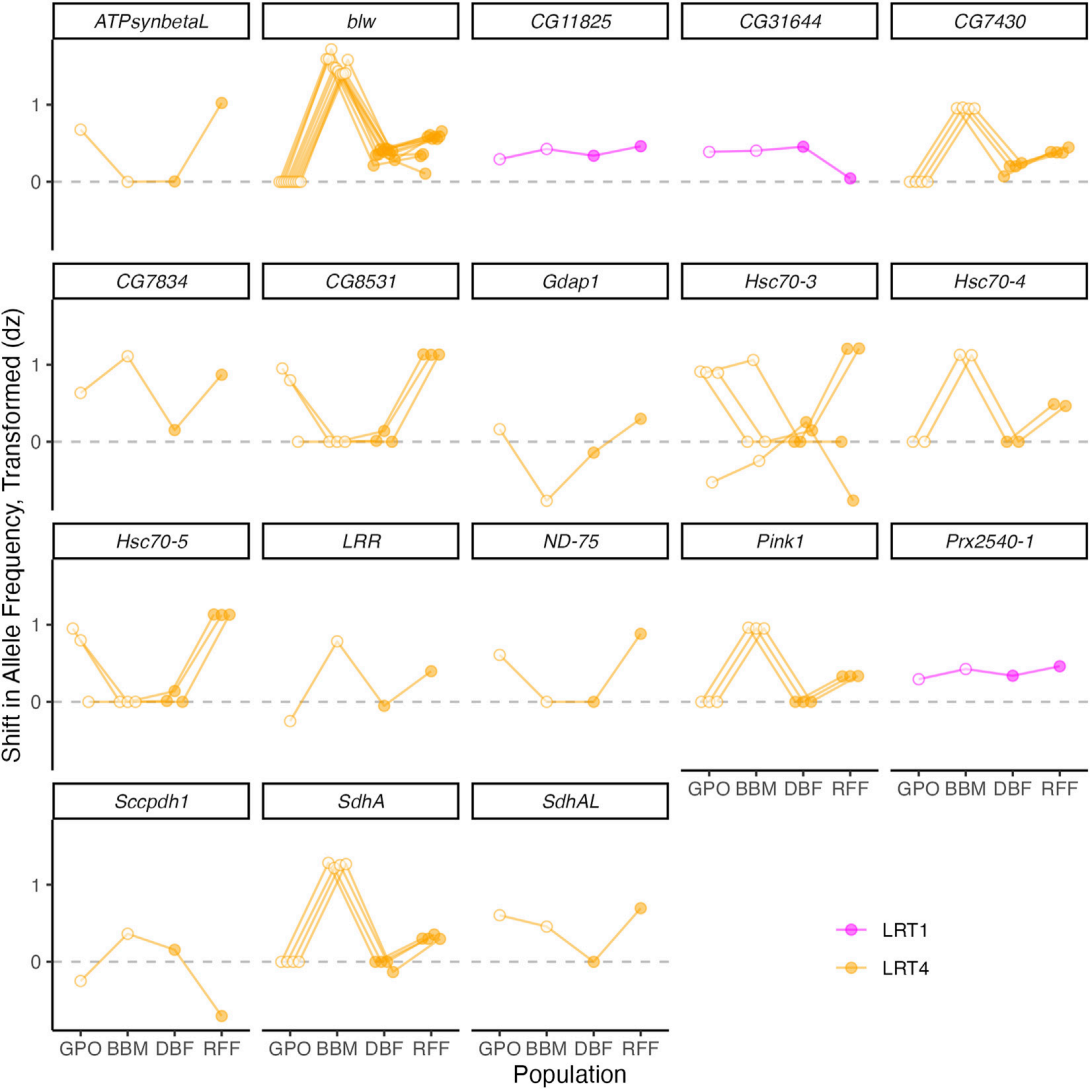
Shift in allele frequency between copper-resistant and control pools of flies from the four wild-derived populations at SNPs that fell within or near genes related to protein folding and mitochondrial function. Positive *dz* values indicate that the reference base is more common in the copper-resistant pool relative to the control pool. Open symbols indicate populations with high copper exposure risk; closed symbols indicate populations with low exposure risk. Note that some symbols perfectly overlap.

Gene ontology analysis of the 238 annotated genes with copper-associated SNPs detected in the wild-derived populations and the DGRP revealed enrichment of genes linked to many categories including those related to protein folding, mitochondrial function, and ATP synthesis (Supplementary Figure S2). The presence of 9 SNPs within three heat shock proteins belonging to the highly conserved Hsp70 family of chaperone proteins contributed to enrichment of these GO categories (Gupta et al., 2010): *Hsc70-3*, *Hsc70-4*, and *Hsc70-5* (The UniProt Consortium et al., 2021) (Figure 6, Supplementary Table S1). Heat shock proteins respond to protein damage caused by oxidative stress following exposure to a diverse set of environmental stressors (Feder and Hofmann, 1999; reviewed in Gupta et al., 2010), and the related gene *Hsp70* has been linked to response to heavy metal stress in bacteria, plants, nematodes, and fish (Williams et al., 1996; Arts et al., 2004; Roh et al., 2006; reviewed in Gupta et al., 2010; Cui et al., 2013; Cui et al., 2019; Zuily et al., 2022) prompting its assessment for use as a bioindicator of pollution, environmental contamination, and climate change (Pyza et al., 1997; Mukhopadhyay et al., 2003; Arts et al., 2004; Roh et al., 2006; Yusof et al., 2022). We recently reported an increase in expression of *Hsc70-3* and *Hsc70-5* in adult *D. melanogaster* females from the DSPR in response to copper stress (Everman et al., 2021). The SNPs that fell within the three Hsc70 chaperone genes had population-specific associations with copper resistance (LRT4, Figure 6). Overall, these patterns suggest that naturally occurring genetic variation in Hsc70 chaperone genes may be under selection for copper resistance to varying degrees across the four populations assessed.

As an essential micronutrient, copper ions act as cofactors for cytochrome c oxidase and super oxide dismutase, and they play a critical role in mitochondrial structure and function (Medeiros and Jennings, 2002; Belyaeva et al., 2012; Zischka and Einer, 2018; Ruiz et al., 2021). However, mitochondria are highly sensitive to excess copper accumulation in cells, and mitochondrial dysregulation of copper is associated with disease in human populations (Huster and Lutsenko, 2007; Zischka and Einer, 2018). Enrichment of GO categories related to mitochondrial function and ATP synthesis were the result of 36 SNPs in or near 16 genes: *blw*, *Prx2540-1*, *CG11825*, *SdhAL*, *ATPsynbetaL*, *CG7430*, *CG7834*, *CG31644*, *ND-75*, *SdhA*, *Hsc70-4*, *Hsc70-5*, *Pink1*, *Gdap1*, *Sccpdh1*, *LRR*, and *CG7834* (The UniProt Consortium et al., 2021) (Figure 6). For all but three SNPs, allele frequency shift was population specific (LRT4). The remaining three SNPs shifted consistently in frequency in the copper-resistant pool across all populations (LRT1) (Figure 6).

Of the 16 genes linked to ATP synthesis, *CG11825* is the only gene that has been previously directly associated with resistance to copper stress (Norgate et al., 2007) (Figure 6). Exposure of the *D. melanogaster S2* cell line to high levels of copper resulted in decreased expression of *CG11825*, and RNAi knockdown increased copper resistance (Norgate et al., 2007). In a previous study, we found a QTL associated with copper resistance in the DSPR that included this gene, suggesting that allelic variation in *CG11825* could influence copper resistance (Everman et al., 2021). Allele frequency shifts (*dz* values) for the variant 65bp upstream of *CG11825* were positive in all four populations, indicating that the alleles present in each population near *CG11825* contribute to copper resistance in a similar manner in the wild-derived populations we examined. Our data do not provide insight into the effect of the upstream SNP on *CG11825* gene function, but regulatory SNPs have the potential to alter phenotype expression; for example, the upstream SNP may influence expression level of *CG11825* in response to copper stress. In a recent study of copper resistance in European populations of *D. melanogaster*, regulatory variation was identified as an important contributor to variation in copper resistance that may be influenced by selection pressure resulting from pollution (Green et al., 2022).

In addition to *CG11825*, the gene *blw* also stands out among the potential candidates associated with mitochondrial function (Figure 6). Ten variants fell within the second and third exons of *blw* and had population-specific effects on copper resistance. *blw* is involved in ATP synthesis, encoding the alpha subunit of mitochondrial ATP synthase (Jacobs et al., 1998). Previously, *blw* mutant flies generated with a transposable element insertion were shown to have higher levels of reactive oxygen species (ROS) resulting in higher levels of oxidative stress (Di Cara et al., 2013). Allelic variation in the promoter region of *blw* has also been associated with increased lifespan in *D. melanogaster* (Carnes et al., 2015), and Garcia et al. (2017) suggested that increased expression of *blw* could increase metabolic activity and the production of ROS. *blw* has not been previously associated with copper metabolism or toxicity. However, copper ions may interact with ATP synthase (Medeiros and Jennings, 2002) and excess copper can lead to decreased energy production and disrupt mitochondrial protein structure and function (Borchard et al., 2018).

Of the remaining genes associated with mitochondrial function, *CG7430*, *TrpRS-m*, and *SdhA* were previously shown to shift in gene expression in response to copper stress in *D. melanogaster* females (Everman et al., 2021), and *Prx2540-1* belongs to a family of peroxiredoxin genes that play a protective role in the response to oxidative stress (Wood et al., 2003). Our results suggest that genetic variation that influences mitochondrial function could be an important contributor to copper resistance even in populations with relatively low copper resistance.

In addition to genes related to mitochondrial function, we detected one SNP within the gene *LRR*, which has been previously linked to response to a neonicotinoid-containing insecticide and is involved in immune response in *D. melanogaster* and bees (*Aphis mellifera*) (Di Prisco et al., 2013). *LRR* is contains leucine-rich repeats, which is a broadly conserved protein domain that facilitates a wide range of responses to immune threats in plants and animals (Ng and Xavier, 2011). *LRR* has not been previously linked to copper resistance or heavy metal response in flies, but genes containing leucine-rich repeats have been linked to arsenic and lead resistance and copper toxicity response in *Arabidopsis thaliana* (Weber et al., 2006; Zhu et al., 2013; Fu et al., 2014). The SNP that fell within *LRR* had population-specific effects on copper resistance.

We did not find an excess of copper-associated genes (based on FlyBase annotation, see Methods) implicated by SNPs that shifted in allele frequency between copper-resistant and control pools. The lack of significant SNPs was not due to a lack of genetic variability in or near copper-associated genes. SNP variants were present in many genes known to be involved in copper homeostasis or metabolism including the five metallothioneins, which are involved in the detoxification of copper, and the copper-responsive transcription factor *MTF-1* (Zhang et al., 2001; Balamurugan et al., 2004) in all four wild-derived populations (Supplementary Table S4). This genetic variation may influence copper resistance in our wild-derived populations with effects that are too small to detect with our experimental design. It is possible that with a larger number of natural replicates of high and low exposure risk populations, SNPs that contribute more moderate improvements to copper resistance could be detected.

Overall, many potential candidate genes were highlighted by our BSA analysis. Several of these potential candidates are especially promising given previous studies in *D. melanogaster* and other species that have associated the genes we identified with responses related to metal or oxidative stress resistance. In particular Everman *et al.* (2021) functionally tested the gene *CG11825* and demonstrated that RNAi knockdown of this gene reduced copper resistance. Similar additional follow up of our novel candidates would be necessary to verify and fully characterize the role they may play in copper resistance.

### 4.3 Copper resistance appears to be subject to both consistent and population-specific selection

Most significant SNPs identified with BSA had population-specific effects on copper resistance (consistent with the LRT4 model), suggesting that the effect of resistance-associated alleles is likely modified by a combination of genetic background and selection regime unique to each of the populations we sampled. The LRT4 model highlights SNPs with alleles that, for example, contribute to increased copper resistance in one population that may not be associated with resistance in another population, or indeed may have an opposite effect on resistance (Figure 2). Many of the candidates identified in the present study are components of larger gene families or pathways (e.g., heat shock proteins (Gupta et al., 2010)) and they interact with other components of these pathways. Genetic variation in interacting genes has the potential to modify the effects of trait-associated alleles through multiple forms of epistasis, a phenomenon that is estimated to be common and itself polygenic (Chandler et al., 2014; Chandler et al., 2017; Goldstein and Ehrenreich, 2021). In yeast, for example, allelic variation in the heat shock chaperone protein *Hsp90* was estimated to modify the phenotypic expression of up to 20% of variable loci in the genome (Jarosz and Lindquist, 2010; Goldstein and Ehrenreich, 2021). Gene by environmental interactions have the potential to further modify these epistatic interactions (Price et al., 2003; Goldstein and Ehrenreich, 2021), and it is likely that epistasis influences copper resistance in our study. However, to assess epistatic interactions, we would need individual-level phenotypes and genotypes, which are not offered in a BSA design. It is also possible that our results are impacted by power; including additional populations with variable copper exposure risk may demonstrate that the predominance of population-specific patterns are characteristic of our focal populations. In the present study, the association between phenotypic variation in copper resistance and proximity to copper pollution combined with population differentiation strongly suggests that the effects of selection for copper resistance are often population-specific and subject to epistatic and environmental interactions.

In addition to the SNPs with significant population-specific effects on copper resistance, we identified 93 significant SNPs with consistent effects on copper resistance in multiple populations, whether the effects were consistent in all populations (LRT1–70 SNPs) or were consistent in the high copper resistance vs. low copper resistance populations (LRT2–23 SNPs) (Table 2). In contrast to SNPs with effects that may be differently modified by selection or background effects, SNPs with consistent effects across populations or classes of populations have the potential to provide insight into evolutionarily strategies for adapting to copper stress. Parallel adaption to copper stress has been previously observed through experimental evolution of yeast (Gerstein et al., 2015). *S. cerevisiae* exposed to elevated copper levels repeatedly evolved mutations in genes related to mitochondrial function as well as by increasing the number of copies of *CUP1-1*, a copper chelator (Gerstein et al., 2015; Hull et al., 2017). Genetic signatures of parallel adaptation in a costal plant species *Silene uniflora* in response to heavy metal pollution from mining activities also point to instances of shared adaptive responses across multiple natural populations (Papadopulos et al., 2021). In each of these cases and in our own study, unique adaptations were predominant; however, the potential for parallel adaptation to heavy metal stress in natural populations based on SNPs with parallel effects warrants further investigation.

## Data Availability

The original contributions presented in the study are publicly available. All sequence data can be found here: https://www.ncbi.nlm.nih.gov/, PRJNA923720 and remaining data is available on FigShare: https://doi.org/10.6084/m9.figshare.22332775.v1.
