# Supplementary material for: The genetic basis of adaptation to copper pollution in Drosophila melanogaster
Source: Front Genet. 2023 Apr 4;14:1144221. doi: 10.3389/fgene.2023.1144221 (PMC10110907; doi:10.3389/fgene.2023.1144221)
Supplement: Supplementary file 1 [file DataSheet2.docx]

Supplementary Material

The genetic basis of adaptation to copper pollution in *Drosophila melanogaster*

Elizabeth R Everman*, Stuart J Macdonald, John K Kelly

*** Correspondence:** Corresponding Author: e.everman@ku.edu

## Supplementary Figures


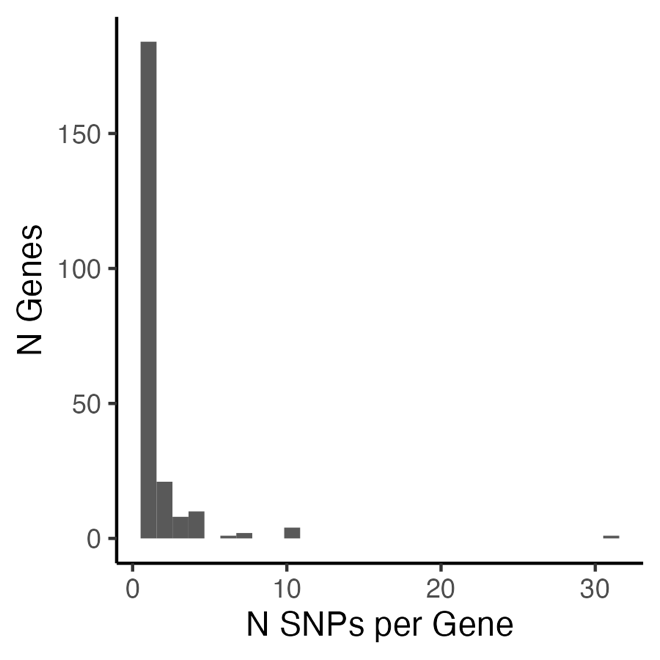


**Supplementary Figure 1.** Multiple SNPs fell within or near 220 genes.


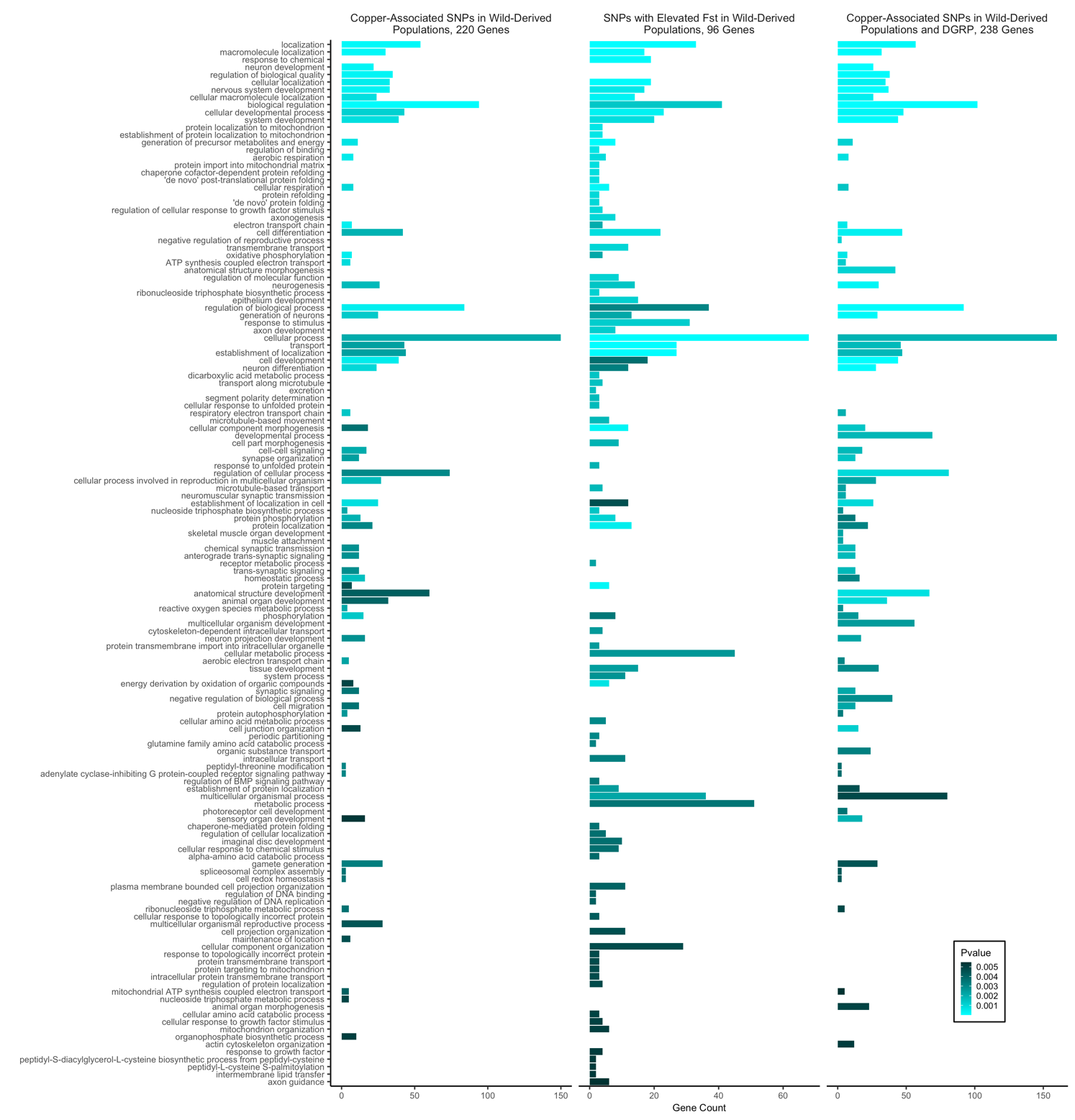


**Supplementary Figure 2.** Gene ontology analysis of genes containing or near SNPs associated with copper resistance in the wild-derived populations. The left panel presents GO enrichment for genes containing one or more significant SNPs associated with copper resistance; the right panel includes all genes implicated by SNPs.


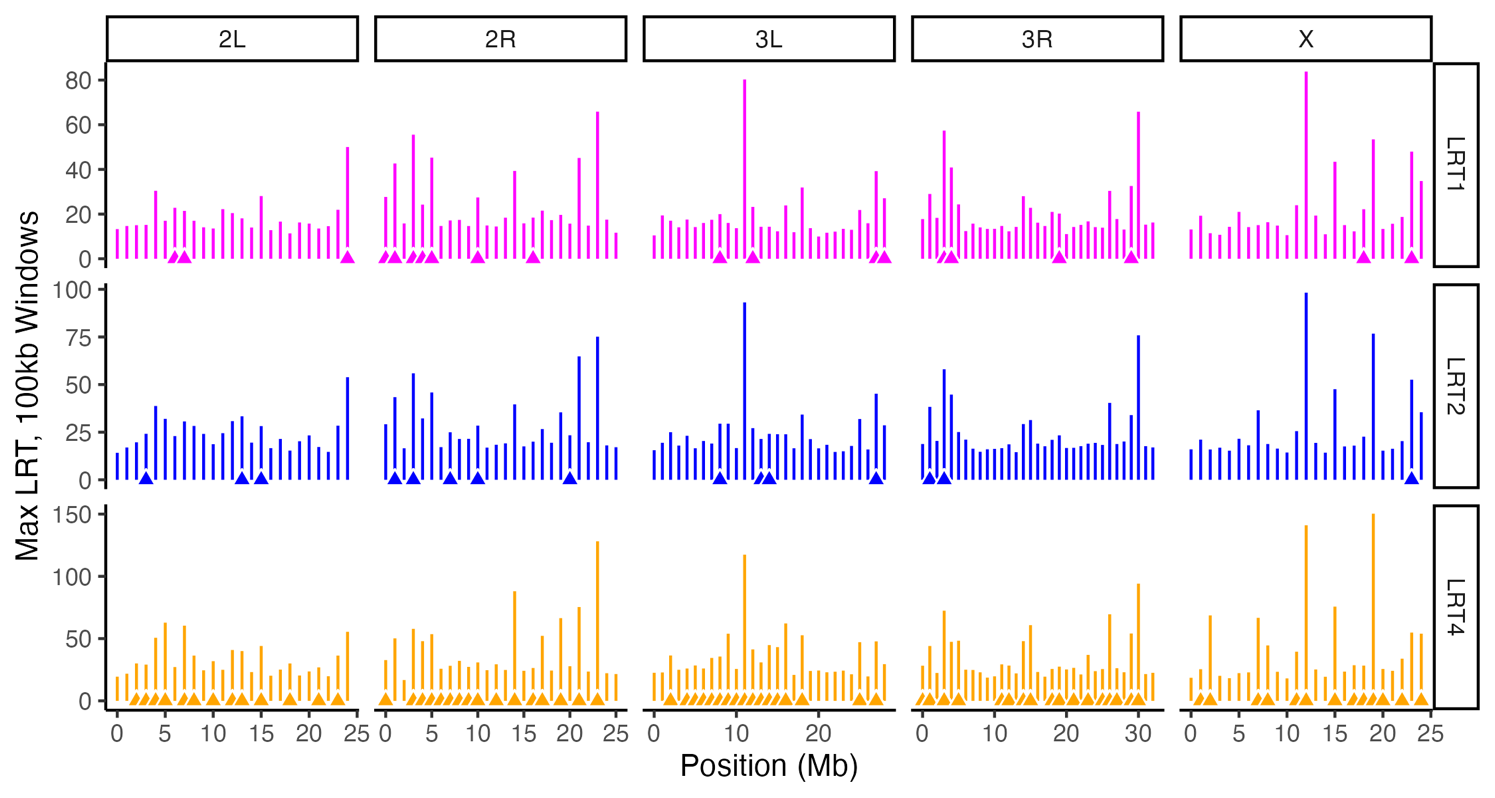


**Supplementary Figure 3.** High max LRT model values determined for 100kb windows distributed along each chromosome arm were more common near centromere regions of chromosomes 2 and 3. Triangle points indicate windows with significant SNPs corresponding to the LRT model indicated in each panel.


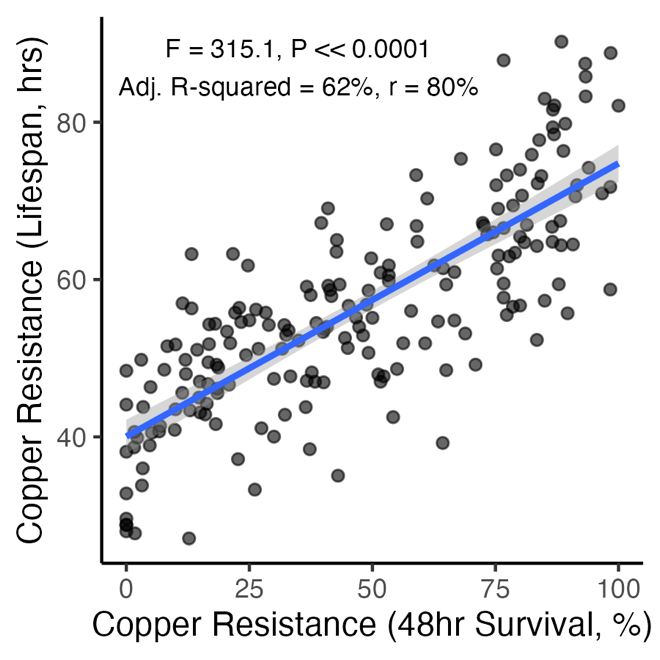


**Supplementary Figure 4.** Average lifespan on copper was correlated with 48-hour copper survival in the DSPR. Copper resistance measured in this study was strongly, positively correlated with our previous measure of copper resistance as survival after 48 hours of exposure (F_1,192_ = 315.1, p << 0.0001, adj R^2^ = 62%, r = 80%; data from Everman et al. (2021)).
